# CD44 Acts as a Signaling Platform Controlling Tumor Progression and Metastasis

**DOI:** 10.3389/fimmu.2015.00154

**Published:** 2015-04-08

**Authors:** Véronique Orian-Rousseau

**Affiliations:** ^1^Institute of Toxicology and Genetics, Karlsruhe Institute of Technology, Karlsruhe, Germany

**Keywords:** CD44, RTK, cancer stem cells, alternative splicing, EMT

## Abstract

Members of the CD44 family of transmembrane glycoproteins emerge as major signal transduction control units. CD44 isoforms participate in several signaling pathways ranging from growth factor-induced signaling to Wnt-regulated pathways. The role of the CD44 family members in tumor progression and metastasis is most likely linked to the function of the various isoforms as signaling hubs. Increasing evidence suggests that these proteins are not solely cancer stem cell (CSC) markers but are directly involved in tumor and metastasis initiation. It is foreseeable that a link between the expression of CD44 isoforms in CSCs and their function as signaling regulators will be drawn in a near future.

The term CD44 designates a large family of transmembrane glycoproteins belonging to the class of cell adhesion molecules. CD44 family members are involved in physiological processes such as hematopoiesis, lymphocyte homing, or limb development [reviewed in Ref. (1)]. All CD44 isoforms are encoded by one single gene located on chromosome 11 in humans and chromosome 2 in mice [reviewed in Ref. (2)]. The exons 1–5 and 16–20 that encode the constant part of CD44 are included in all CD44 isoforms. Exons 6–15 that encode the variant exons v1–v10 are either completely excluded as in CD44s or are included in various combinations within the CD44 ectodomain, giving rise to the CD44 variant isoforms (CD44v). Of note, exon v1 is not expressed in human cells due to the presence of a stop codon. The heterogeneity of the CD44 family is further increased by several additional modifications including N- and O-glycosylations.

## CD44 Expression

**Figure 1:**
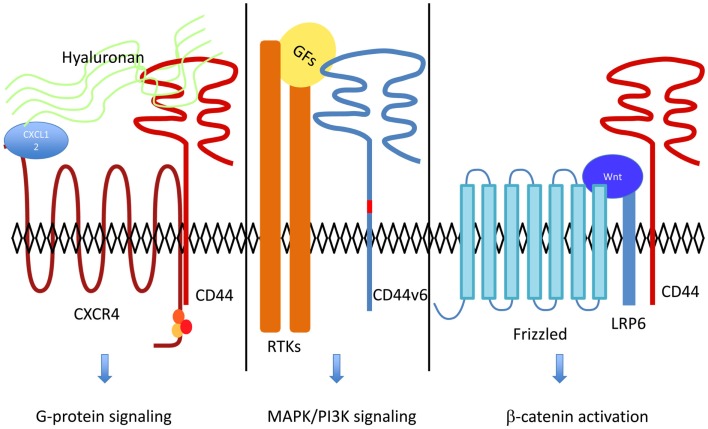
**CD44 acts as a co-receptor for several cell surface receptors including RTKs, G-protein coupled receptors, and LRP6**. Hyaluronan binding to CD44 increases CXCL12-induced CXCR4-G Protein signaling (18). RTKs activation by their ligands [growth factors (GFs) such as HGF or VEGF] and downstream signaling are dependent on CD44v6 [reviewed in Ref. (11)]. In the Wnt-β-catenin pathway, LRP6 recruitment of CD44 leads to β-catenin activation and translocation to the nucleus (3).

CD44s is expressed in nearly all tissues whereas the expression of CD44v isoforms is restricted to specific cell types [reviewed in Ref. (1) and table in Ref. (3)]. In human skin, the longest CD44 isoform containing the variants v2–v10 can be detected. Expression of CD44 variants on normal lymphohematopoietic cells is generally low. T lymphocytes activation by antigen or by mitogen leads, however, to transient expression of variant isoforms such as CD44v6 (4, 5). CD44v6 isoforms are also found in proliferative tissues such as the skin or the intestine (Figure 1).

Overexpression of various CD44 isoforms has been found in many types of human tumors. A comprehensive review was published by Naor et al. (6). In colorectal cancer, specific CD44 isoforms are expressed according to the progression of the disease (7). Isoforms containing the v5 exon could already be detected in early adenomas. The expression of v6-containing isoforms was detected in early and advanced polyps as well as in invasive carcinomas where it correlated with the Duke stage (7). Interestingly, a cell-specific splicing was described in the intestine (8). Remarkably, the lgr5^+^ stem cells were shown to be CD44v4-v10^+^ whereas transit-amplifying cells exclusively expressed CD44s or smaller CD44v isoforms but not CD44v4-v10. Human cryptic foci corresponding to microadenomas retaining a stem cell program express CD44v4-v10 as well but not CD44s. In breast cancer, expression of CD44v6 was detected in intraductal carcinoma and was related to tumor invasion, metastasis, and pathological grade (9). CD44s and CD44v6 were shown to be up-regulated in pancreatic cancer adenocarcinoma (10). CD44s expression but not CD44v6 was associated with worse overall survival. In head and neck cancer patients, high expression of CD44v6 prompted the development of a CD44v6 antibody for clinical trial phase I [reviewed in Ref. (2)].

## CD44 Acts as a Signaling Hub

In some cases, the role of CD44 in tumor progression could be linked to its function as the main receptor for hyaluronan (HA), a major component of the extracellular matrix (ECM) [reviewed in Ref. (11)]. This was, for example, the case in breast cancer (12). The question remains whether other molecular functions of CD44 drive its involvement in specific types of cancers. Accumulating evidence demonstrate that CD44 acts as a signaling hub controlling cell surface receptors of very diverse structure and function, e.g., CD44v6 isoforms act as co-receptors for RTKs such as Met, Ron, or VEGFR-2 [reviewed in Ref. (11)]. The function of CD44v6 for these RTKs is a twofold. On one hand, the CD44v6 ectodomain drives the activation process of the RTK. On the other hand, the cytoplasmic domain recruits ezrin–radixin–moesin (ERM) proteins together with the cytoskeleton in order to promote signaling from the RTK. CD44v6 and Met could be found in a complex with the Met-ligand HGF (13). Furthermore, Met internalization was shown to depend on CD44v6 (14). Intriguingly, the function of the CD44v6 isoform for VEGFR-2 is very similar to the CD44v6/Met pair. VEGF-induced VEGFR-2 activation depends on CD44v6 and in addition VEGFR-2-induced signaling requires the link of CD4v6 to ERMs and to the cytoskeleton (15). Moreover, CD44v6 and VEGFR-2 could be found in a complex, which was not inducible by VEGF. Most strikingly, CD44v6-dependent Met (16) and VEGFR-2 activation (15) could be blocked by small peptides containing a CD44v6 exon sequence; these peptides inhibited the angiogenesis of pancreatic tumors (15).

A participation of various CD44 isoforms in the activation of other RTKs (EGFR, PDGFR, or FGFR) was reviewed in Ref. (11). For example, CD44v3 heparan-sulfated isoforms were shown to act as a recruitment platform for metalloproteinases like MMP-7 (or matrilysin) involved in the maturation of HB-EGF and subsequent activation of ErbB4 (17).

A completely different receptor, namely, CXCR4 also appears to be dependent of CD44 (18). This time the binding of HA to CD44 is instrumental for this co-operation. High-molecular weight HA binding to CD44 augmented the activation of CXCR4 by its ligand CXCL12 whereas small HA fragments completely abrogated the CXCL12-induced CXCR4 signaling. CD44 and CXCR4 were found in a CXCL12-dependent complex regulated by HA. Interestingly, this interaction appears to be crucial for angiogenesis.

More recently, the Wnt-target gene CD44 (19) was shown to regulate Wnt signaling (3). Overexpression or downregulation of various CD44 isoforms modulated positively or negatively, respectively, Wnt activity. Downregulation of CD44 expression inhibited activation of β-catenin and subsequent translocation to the nucleus. The role of CD44 in Wnt signaling was independent of its binding to HA. In contrast, the binding of the CD44 cytoplasmic domain to ERMs was instrumental. Epistasis experiments revealed a function of CD44 at the level of LRP6 and both LRP6 and CD44 could be found in a Wnt-inducible complex. The function of CD44 for LRP6 is dual. On one hand, CD44 controls LRP6 activation, a Wnt-dependent event. On the other hand, CD44 is involved in LRP6 maturation, a Wnt-independent step. Indeed, downregulation of CD44 expression lead to a lack of LRP6 membrane expression and to an inhibition of Wnt-induced LRP6 phosphorylation. Downregulation of CD44 in the central nervous system of *Xenopus laevis* embryos impaired expression of Wnt-regulated genes such as eng-2 and tcf-4. CD44 is therefore able to act as a Wnt-target gene as well as a Wnt regulator.

## Alternative Splicing of CD44 Isoforms in Tumor Progression

Since CD44s and CD44v isoforms are involved in tumor progression and metastasis alternative splicing of CD44 seems to be a decisive event controlling the progression of cancer. Several years ago, a mini-gene construct was used to investigate exon v5 alternative splicing and the relevance of signal transduction for this process was shown (20). The nuclear RNA-binding protein Sam68 was shown as a decisive factor controlling CD44 splicing (21). This splice regulator was under the direct control of Erk and consequently under control of Ras confirming the involvement of growth factor signaling in the regulation of CD44 alternative splicing. Moreover, a positive feedback loop in which Ras signaling-induced CD44v6 splicing was unraveled. In turn, CD44v6 promoted late Ras signaling, which was shown to be important for cell cycle progression (22).

The regulation of CD44 alternative splicing during tumor progression is still not completely unraveled. Conversely, the steps in tumor progression regulated by alternative splicing of CD44 still need to be defined. Some indications came from studies on epithelial–mesenchymal transition (EMT). EMT is a process by which epithelial cells loose their polarity, gain invasive properties, and acquire mesenchymal features. On one hand, EMT was shown to induce a CD44^+^ phenotype (23). On the other hand, a shift from CD44v to CD44s was essential for cells to undergo EMT as well as for the formation of breast tumors. In this case, a decreased expression of the splicing factor epithelial splicing regulator 1 (ESPR1), which promotes the switch to CD44v isoforms was critical for EMT. In other studies, the heterogeneous nuclear ribonucleoprotein M (hnRNPM) was identified as an essential splicing regulator involved in TGFβ-induced EMT (24). hnRNPM-mediated CD44 exon skipping was induced through inhibition of ESPR1 function and was essential for breast cancer metastasis.

In contrast to the above described studies, colonization of the lung by 4T1 mouse breast cancer cells was shown to be dependent on the switch from CD44s to CD44v isoforms (25). This time the knockdown of the EPSR1 protein led to the reduced cell surface expression of the Na^+^-independent cystine transporter xCT and suppressed lung colonization.

In the same line, the activation of the Wnt pathway, a central player in EMT, induced CD44v6 expression in colorectal cancer cells [reviewed in Ref. (26)] as well as in breast cancer cells (27).

## Function of CD44 on Cancer Stem Cells

A link between EMT and stemcellness was demonstrated using immortalized mammary epithelial cells (23). EMT was shown to generate cells with many of the properties of self-renewing stem cells. These cancer stem cells (CSCs) that have the ability to seed a tumor were shown to be CD44^+^. CD44 is also expressed on several other types of CSCs including pancreatic and colorectal CSCs [reviewed in Ref. (28)]. However, little is known on the molecular function of CD44 in these CSCs. A link between the presence of specific CD44 isoforms on CSCs and their function as co-receptors might exist. Several years ago, CD44 and EpCAM were described as robust markers of colorectal CSCs (29). More recently, the CD44v4-v10 isoform was detected on lgr5^+^ stem cells in the intestinal crypts. The presence of this CD44 isoform on colorectal cancer cells was linked to tumor progression in Apc^Min/+^ mice. The function of CD44 in colorectal cancer might be due to its role as a modulator of Wnt signaling (3) or as a Wnt-target gene since one partner of CD44v6, namely the Met RTK, is also over-expressed in colorectal cancer (30). Met overexpression could be detected already in dysplastic aberrant crypt foci, one of the earliest lesions in colorectal cancer. Similarly to CD44, Met expression seems to be controlled by Wnt. Therefore, collaboration between the Wnt-target genes Met and CD44v6 might be required for the progression of colorectal cancer. This idea was further strengthened by the finding that the metastatic potential of colorectal spheres orthotopically injected in the mouse cecum was abrogated by depletion of CD44v6 and Met (31). Furthermore, in colorectal CSCs, the expression of CD44 is controlled by several cytokines including HGF by activating the Wnt pathway thereby promoting migration and metastasis (31).

Interestingly, circulating primary luminal breast cancer cells containing a population of metastasis-initiating cells (MICs) express among other markers Met and CD44 (32). However, whether any interplay between Met and CD44v6 is necessary for the metastatic potential of these MICs is not yet clear.

CD44 was suggested as a marker on acute myeloid leukemia (AML) leukemic stem cells (LSCs) [reviewed in Ref. (28)]. A mAb (H90) mediating CD44 ligation inhibited AML–LSCs homing to the bone marrow and their engraftment. The same antibody was shown to abrogate adhesion of AML CD34^+^CD38^−^ cells to HA suggesting that CD44 binding to HA is involved in the homing process and growth in the bone marrow. Moreover, the interaction between CXCR4 on leukemic cells and CXCL12 in the niche is needed for the homing and growth of LSCs (33). Taken together, these data strongly suggest that the molecular function of CD44 and HA in CXCL12-induced signaling (18) might be involved in AML.

## Outlook

For long, the role of CD44 in tumor progression and metastasis remained unclear. Many lines of evidence indicate that CD44 organizes a signaling platform at the cell surface by acting as a co-receptor for various types of cell surface receptors (Figure 1). In addition, large amount of data show that CD44 is a bone fide CSC marker. Understanding whether these two aspects are linked will require a better knowledge of the functions of CD44 isoforms at the molecular level and the systematic identification of specific CD44 isoforms expressed on CSCs. A CD44-based therapy targeting CSCs will certainly benefit from specific tools blocking the co-receptor function of CD44 for cell surface receptors.

## Conflict of Interest Statement

The author declares that the research was conducted in the absence of any commercial or financial relationships that could be construed as a potential conflict of interest.
